# Dual Frequency Head Maps: A New Method for Indexing Mental Workload Continuously during Execution of Cognitive Tasks

**DOI:** 10.3389/fphys.2017.01019

**Published:** 2017-12-08

**Authors:** Thea Radüntz

**Affiliations:** Mental Health and Cognitive Capacity, Work and Health, Federal Institute for Occupational Safety and Health, Berlin, Germany

**Keywords:** mental workload, electroencephalography, biomedical signal processing, pattern recognition, state monitoring

## Abstract

One goal of advanced information and communication technology is to simplify work. However, there is growing consensus regarding the negative consequences of inappropriate workload on employee's health and the safety of persons. In order to develop a method for continuous mental workload monitoring, we implemented a task battery consisting of cognitive tasks with diverse levels of complexity and difficulty. We conducted experiments and registered the electroencephalogram (EEG), performance data, and the NASA-TLX questionnaire from 54 people. Analysis of the EEG spectra demonstrates an increase of the frontal theta band power and a decrease of the parietal alpha band power, both under increasing task difficulty level. Based on these findings we implemented a new method for monitoring mental workload, the so-called Dual Frequency Head Maps (DFHM) that are classified by support vectors machines (SVMs) in three different workload levels. The results are in accordance with the expected difficulty levels arising from the requirements of the tasks on the executive functions. Furthermore, this article includes an empirical validation of the new method on a secondary subset with new subjects and one additional new task without any adjustment of the classifiers. Hence, the main advantage of the proposed method compared with the existing solutions is that it provides an automatic, continuous classification of the mental workload state without any need for retraining the classifier—neither for new subjects nor for new tasks. The continuous workload monitoring can help ensure good working conditions, maintain a good level of performance, and simultaneously preserve a good state of health.

## 1. Introduction

Advanced information and communication technology, highly interactive work environments, and work assistance systems impose increasingly high demands on our cognitive capacity and on the ability to cope with mental workload. Although one main goal of information and communication technology is to simplify work, there is growing consensus concerning the negative consequences of inappropriate workload on human's health and the safety of persons. Reports show that employees are complaining more frequently about high mental workload and stress (Kompier and Kristensen, 2001; NIOSH, 2002; Landsbergis, 2003; Lohmann-Haislah, 2012). At the same time, increased automation can be also associated with monotonous tasks, a decrease in arousal, and underload (Hacker and Richter, 1984; Parasuraman et al., 1993, 1994; Debitz et al., 2003; May and Baldwin, 2008).

Optimization of work conditions in human-machine systems is a big challenge. Under optimized work conditions we await performance at its best whilst simultaneously preserve employee's health. In this context, a reliable and objective method for capturing mental workload continuously is absolutely essential. Hence, the long-term goal is to develop a method to be able to recognize critical states like high and low workload.

### 1.1. Theoretical background

Mental workload has a long history in the psychological literature. However, different authors refer to it with different concepts and there is neither a common definition of mental workload nor a clear methodology for measuring it. The concept of mental workload was first introduced in the 1940s (Bornemann, 1942) with the aim of optimizing human-machine systems. Although various definitions have followed since then, the core of the concept is the relation between task demands and personal capacity. According to Kahneman (1973) cognitive resources for human information processing are limited and hence the amount of task demands placed on a person's limited resources is equivalent to mental workload. Similar definitions are given by Eggemeier et al. (1991) where “Mental workload refers to the portion of operator information processing capacity or resources that is actually required to meet system demands.” Mental workload is assumed in Wickens (2002) to describe the “relation between the (quantitative) demands for resources imposed by a task and the ability to supply those resources by the operator.” In Xie and Salvendy (2000) time is also included as an important factor and it is suggested that “mental workload is the amount of mental work or effort necessary for a person or group to complete a task over a given period of time.”

Considering the enormous historical body of literature and in order to put our work on the footing of a consistent theoretical background mental workload in this article is defined in line with the definition of mental strain according to the ISO standard in International Organization for Standardization (1991). There mental strain is specified as “the immediate effect of mental stress within the individual (not the long-term effect) depending on his/her individual habitual and actual preconditions, including individual coping styles.” This definition reflects cognitive and emotional processes in the human brain that are characterized by neurophysiological changes in the central nervous system. Furthermore, these processes also imply a shift to catabolic activity within the autonomic nervous system (Fairclough et al., 2005). This reaction represents a compensatory strategy to preserve performance under increased demands and stressors (Hockey, 1993, 1997).

### 1.2. Assessment of mental workload

Assessment of mental workload can be done by subjective and objective methods. Subjective methods ask the subject for a rating by mean of questionnaires. Examples for objective methods are performance measurements and psychophysiological measurements.

Subjective methods consist of relatively low data acquisition effort and high user acceptance. They would offer a straightforward solution to register workload if they were not sensitive to subjective distortion. They are problematic because firstly individuals are expected to provide a certain kind of answer and secondly the experienced workload took place at some time in the past. Additionally, there might also exist discrepancies due to the subjects not having understood the questionnaire's items or due to an inability to introspect. Importantly, they do not allow for fine-grained temporal sampling, e.g., on the time scale of seconds, without clearly reducing user acceptance. Furthermore, surveys can alter the current workload state, e.g., if during a monotonous task the subject becomes activated by answering questions.

Although performance measurements offer an objective way to define workload, we have to be aware of their main issue. It is a fact that in order to cope with increased demands individuals adapt their behavior. They invest more effort and experience more mental workload to maintain performance at the same level. In this sense performance measurements are not sensitive to the increase of workload while subjective ratings and physiological indicators may reflect changes. However, in cases where individuals change their task goals and accept a lower level of performance or even give up (Meijman and O'Hanlon, 1984), performance measures will indicate a decrease (hence increased workload) while physiological measures may remain unchanged or show decreased workload. However, to capture high mental workload that could affect individual's health we have to concentrate on situations where individuals keep engaged to the task and try to maintain their performance even though task demands increase and they have to invest more effort. Performance measurements are thus insufficient.

Objective methods as the analysis of biosignals offer the capability to continuously determine mental workload. Eligible biosignals for workload registration are e.g., the EEG, cardiovascular parameter as well as ocular data. The signals react differently by different kinds of workload (e.g., emotional, physical, or mental workload) but also between different subjects. User acceptance varies with respect to the complexity of the registration system constituting their main issue. However, recent developments in the field of mobile technology promise small, lightweight, and wireless systems. In addition, a main advantage of biosignals is that they can be obtained continuously, a subject can hardly manipulate them, they do not alter subject's current workload state, and they can be obtained on-the-fly during task execution.

Previous studies indicated that several psychophysiological variables respond in a more or less predictable way to cognitive effort, e.g., heart rate variability (Veltman and Gaillard, 1996; Mulder et al., 2002), blood pressure (Veltman and Gaillard, 1996), the P300 from the EEG (Ullsperger et al., 1988), pupil diameter (Beatty, 1982), and adrenaline concentration (Frankenhaeuser et al., 1980).

### 1.3. Neural indexing of mental workload

In our article we focus on power spectrum analysis of the electroencephalogram. The reason is that features of brain activity have a unique potential for achieving mental workload registration. For the sake of completeness, we have to mention that there are also a number of studies that try to estimate mental workload by use of neurophysiological components from event related potentials (ERPs) (Brouwer et al., 2012; Roy et al., 2016). However, the great advantage of spectral features against ERPs is that they are less intrusive and more practical regarding real-life applications. Spectral features allow continues monitoring of mental state without any need for releasing stimuli and triggering their appearance time.

There is a lot of research work with the aim to establish a reliable method for mental workload estimation by use of spectral features from the EEG (Brouwer et al., 2015). Some researchers use ratios of the spectral power frequency bands (Brookhuis and de Waard, 1993; Pope et al., 1995; Prinzel et al., 2001), try to define thresholds for them, or work with single power spectra and classification algorithms (Berka et al., 2007; Heger et al., 2010; Dijksterhuis et al., 2013). In general, threshold values are problematic because they are not commonly applicable to different subjects. Individually, i.e., for each single person, trained models for EEG classification offer an acceptable solution (Brouwer et al., 2012). Therefore, classifiers have to be trained for each subject and also in regard to the task set and its different workload levels. After that the model can be used for estimating the subject's mental workload during the particular task. This individual adjustment and retraining of the classifier to subject and specific task is time-consuming and cumbersome. However, many years of investigation in the field of mental workload, experience with EEG analysis, and the results of numerous EEG studies (Gundel and Wilson, 1992; Wilson, 2001, 2002b; Berka et al., 2007; Kohlmorgen et al., 2007; Lei et al., 2009; Lei and Roetting, 2011) form the solid basis for the development of our method for mental workload monitoring.

Our aim is to obtain individual classification of a subject's mental state independent of a specific task. Furthermore, retraining of the classifier should not be necessary, neither for new subjects nor for new tasks. For achieving these objectives, we developed a new method for continuous mental workload classification based on the so-called Dual Frequency Head Maps (DFHM). These DFHM rely on personalized spectral features and their spatial occurrence. They are classified with support vector machines (SVMs) trained by means of established knowledge linked to the variability of the spectral features due to mental demands that have been reported in EEG research over the past 50 years. In particular, increased mental workload is mainly associated with EEG variations in the spectral power of the theta band at frontal sites (Inouye et al., 1994; Gevins et al., 1997; Smith et al., 2001; Sammer et al., 2007) and of the alpha band at the parietal area (Gevins et al., 1997; Smith et al., 2001; Wilson, 2002a; Wilson and Russell, 2003). The theta band is considered to be a reliable parameter which is enhanced with increasing task difficulty (Gevins et al., 1998; Jensen et al., 2002), whereas the alpha band seems to be less reliable with respect to the decrease which is normally expected (e.g., Hagemann, 2008). Some studies have linked this behavior to different kinds of attention (internal vs. external) or other task requirements (Klimesch, 1999; Kelly et al., 2006).

The conducted experiments are described in section 2, starting with the description of the cognitive task battery we implemented for data acquisition. The subsequent section 3 introduces the new method and the proceeding for its evaluation while the results from the statistical analysis are presented in section 4. Furthermore, the empirical validation of the DFHM method on a new sample set and task are described in section 5. Finally, the results are discussed in section 6 and conclusions about limitations and impact of the study are drawn.

## 2. Materials and experiments

### 2.1. Tasks

In order to develop a method based on well-grounded theory to quantify mental workload by means of the EEG, we specifically put together an appropriate set of tasks with reduced confounding factors for inducing different levels of workload. According to Berka et al. (2007) this set should consist of relatively pure tasks that induce cognitive state changes by manipulation of task demands. A method created using such tasks is meant to be more valid when used in more complex operational situations. This is due to the fact that mental states involved in complex tasks can be broken down into fundamental cognitive processes such as attention, working memory, and executive function resources (Berka et al., 2007). Latter are necessary to cope with operational environments. Hence, we established a link to the state-of-the-art research on working memory processes and executive functions.

Baddeley's theory (Baddeley, 1986, 2000, 2012) offers a starting point of a central attentional control system that was modeled following the supervisory attentional system by Norman and Shallice (1986). According to this there exist on the one hand situations that allow for schema-based processing and require little attentional control. Responses in these cases run quite automatically because they are based on well-learned schemata in long-term memory. On the other hand there exist situations that cannot be handled by schemata and demand action by a central attentional control system to inhibit automatic responses. Furthermore, they require the selection of task adequate, new responses. In Miyake et al. (2000) three executive functions are defined to be needed during non-automated processing: inhibition, shifting, and updating.

Within this framework we selected nine cognitive tasks (0-back, 2-back, Sternberg, serial Sternberg, Stroop, switch-PAR, switch-NUM, switch-XXX, AOSPAN) for our task battery. They overlap somewhat with regard to task demands but differ with regard to the executive function addressed (Table 1). Their implementation was realized with the E-Prime application suite.

**Table 1:** The tasks and their preferential workload source.

**Task**	**Duration**	**Working**	**Executive functions**			
	**[min]**	**memory**	**Updating**	**Inhibition**	**Shifting**	**Multitasking**
0-back	5	(x)				
2-back	5	x	x			
Sternberg	10	xx				
ser. Sternberg	10	xx	x			
Stroop	5	x		x		
switch-PAR	5	x				
switch-NUM	5	x				
switch-XXX	10	x			x	
AOSPAN	20	xxx	x		x	x
rest start	3					
rest end	3					

*The claiming intensity of each executive function is indicated by the number of Xs*.

The 0-back task represents an easy task, where the subject will have to press the mouse button if the presented letter is “X.” In the 2-back task the subject will be asked to press the mouse button if the presented letter is the same as the next to last letter seen (Kirchner, 1958; Gazzaniga et al., 2013). Hence, subjects are updating their working memory continuously.

During the Sternberg task (Sternberg, 1966) a set of two or six letters at a time appears on the screen. After an interval of 1,000 ms, a test letter is presented. Subjects will be instructed to press the red mouse button if the test letter has been shown before and the green mouse button otherwise. Furthermore, they are instructed to not read the letters aloud.

The serial Sternberg task (Raghavachari et al., 2001) is a combination of the n-back task with the Sternberg task. Hence, it is a working memory task with updating requirements. Single letters appear serially on the screen, followed by a test letter at the end of the sequence. Subjects will be instructed to press the red mouse button if the test letter has been shown before and the green mouse button otherwise. The number of the presented letters varies between two and six and the subjects are not allowed to read the letters aloud.

The Stroop task (Stroop, 1935) is an inhibition task. Differently colored words appear on the screen one at a time. The subject is asked to press the mouse button (yellow, green, red, and blue) that matches the font color, ignoring the meaning of the word.

The switch task consists of two easy single blocks and a more demanding mixed block (Gajewski et al., 2012). In the single blocks participants have to classify digits according to their numeric value (NUM) or their parity (PAR). In the mixed blocks the participants have to memorize the switching rule between these two conditions (NUM, NUM, PAR, PAR, NUM etc.) and process the digits differently every second time.

The AOSPAN task is administered as a demanding dual task in the version developed by Unsworth et al. (2005) and was translated and adapted by the author. Subjects are asked to memorize letters in the order presented while simultaneously solving math problems. The math problem requires to click as soon as they know the answer. After the click a number is presented and they have to decide if it is the right answer to the problem. Then a letter to be memorized is shown. At the end a recall slide is presented asking them to select the letters shown in the correct order. Finally, they get feedback about both their memory and math performance. Furthermore, the subjects are instructed to keep the percentage number indicating their math performance above 85%.

For all tasks the subjects are instructed to work as quickly and accurately as possible. A more detailed description of the tasks including slide presentation times, number of trials, and number of targets can be found in Radüntz (2016).

Performance evaluation for all tasks was done by analysis of individual accuracy rates. In case of the AOSPAN task, the accuracy rates were calculated from the sum of correctly recalled letters from only the items in which all characters were recalled in correct serial order.

### 2.2. Procedure

The EEG investigation was performed in a shielded lab under well-controlled laboratory conditions at the Federal Institute of Occupational Safety and Health in Berlin. We asked subjects to participate in a 1-day experiment where they had to complete the above-mentioned cognitive tasks.

The experiment consisted of a training procedure and the main test. The training procedure served to familiarize the subjects with the cognitive tasks and created similar starting conditions for all participants. The training tasks were the same as those of the main experiment but shorter in duration. They were repeated until the subject reached an accuracy index of at least 80%. In that way we aimed to control the effect of task demands on the registered mental workload independent from learning effects. Only the training of the AOSPAN task was performed during the main experiment. It took place directly before the actual task as described in Unsworth et al. (2005). The math practice of the task aimed to calculate for each person how long they need to solve the math operations. Each individual's mean (plus 2.5 SD) was used during the main AOSPAN task as a time limit for the math problem.

The main experiment started after a short break subsequent to the training. It was controlled remotely through a remote desktop connection, an intercommunication system, and a video monitoring system. The tasks were presented to the subjects in a counterbalanced order. The duration of each task is presented in Table 1. The participating subjects needed on average 90 min to complete all tasks. Physiological signals were digitally recorded only during the main experiment. They were also measured during resting periods (with eyes open) of about 3 min at the beginning and the end of the main experiment to serve as reference measurements.

### 2.3. Subjective ratings

We used the NASA-TLX questionnaire (Hart and Staveland, 1988) in a computerized version for capturing subjective workload. Subjects were asked to first rate the workload sources after each task during the training phase. That was done by 15 pairwise comparisons of the NASA-TLX's six workload dimensions: mental demand, physical demand, temporal demand, performance, effort, and frustration. Analysis of subjects' pairwise choices indicates the perceived importance of each dimension and hence its contribution to the task specific workload. Furthermore, the paired comparisons enable us to gain individualized weightings of the subscales. The second part of the NASA-TLX questionnaire was conducted during the main experiment. Ratings of each workload dimension within a 100-point range were done after each task by clicking on a 5-point step box with an optical mouse.

### 2.4. Subjects

Fifty seven people in paid work and between the ages of 34 and 62 years participated in our study. Hence, the individually occurring mental workload was expected to vary due to the high variability of the sample in respect to cognitive capacity and age. Three persons had to be excluded from further analysis because they did not complete all cognitive tasks. Hence our final sample set consists of 54 subjects (31 female, 23 male, mean age 41).

All of the investigations acquired were approved by the local review board of our institution and the experiments were conducted in accordance with the Declaration of Helsinki. All procedures were carried out with the adequate understanding and written consent of the subjects.

## 3. Methods

Digital signal processing and all calculations were done with MATLAB.

### 3.1. EEG and the new method of DFHM

#### 3.1.1. Pre-processing

**Figure 1:**
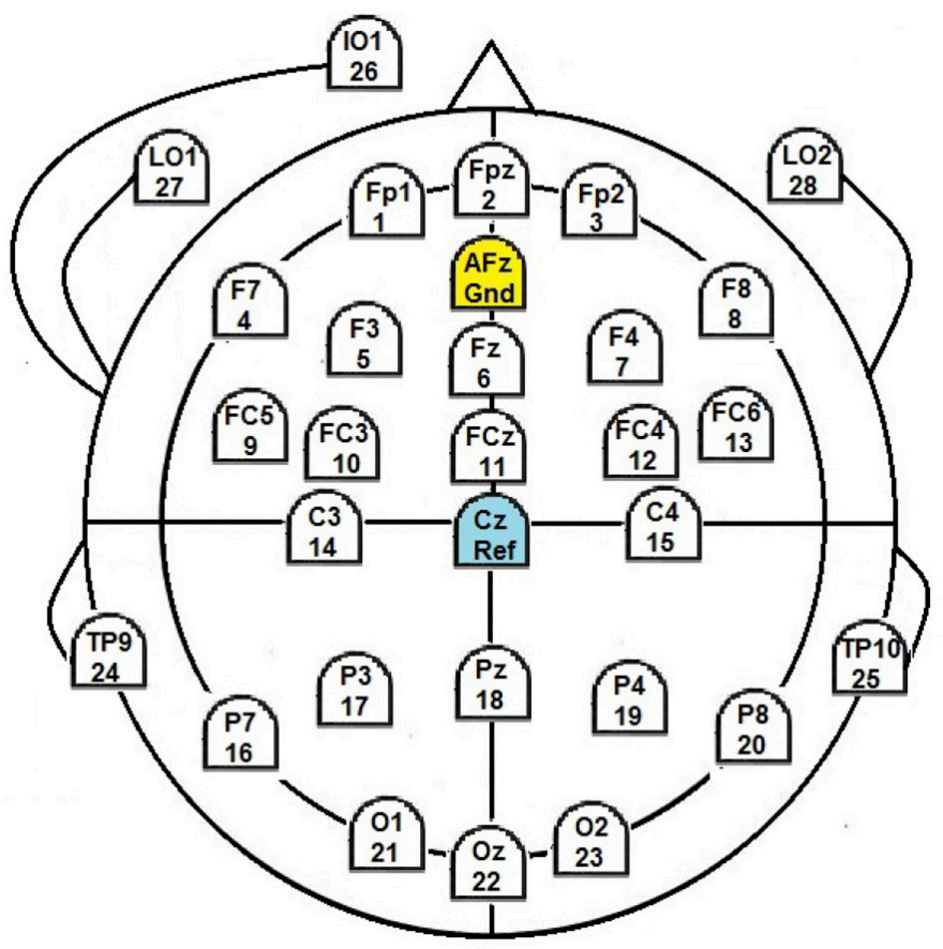
EEG-layout montage used.

Twenty five passive electrodes were placed at positions according to the 10–20-system for capturing the EEG (Figure 1). Registration was carried out with a sample rate of 500 Hz and with reference to electrode Cz. For signal recording we used an amplifier from BrainProducts GmbH and their BrainRecorder software.

The recorded EEG is windowed with a Hamming function and filtered with a bandpass filter (order 100) between 0.5 and 40 Hz. Independent component analysis (ICA, Infomax algorithm, Makeig et al., 1996) is applied to the signal and the independent components calculated are automatically classified as either an artifact or signal component (Radüntz et al., 2015, 2017). The signal components are projected back onto the scalp channels and constitute the artifact-free EEG. In the following, the artifact-free EEG is transformed to average reference and cut into segments of 10 s length, overlapping by 5 s. Workload relevant frequency bands (theta: 4–8 Hz, alpha: 8–12 Hz) are computed over the segments using the Fast Fourier Transformation (FFT).

#### 3.1.2. Z-scores and generation of DFHM

The spatial fusion of the frontal theta band power with the parietal alpha band power for each segment constitutes one of the main concepts in our method. For this, we apply a theta-bandpass filter to the signals of the frontal electrodes and an alpha-bandpass filter to the signals of the parietal electrodes. Next, for reducing individual variation, we calculate for each participant the z-scores of theta and alpha band power of each electrode. The z-score represents the distance between a value and the mean of the data in units of the standard deviation.

For the z-score calculation, we use beforehand calculated individual means and standard deviations for the two band powers of each channel. These descriptive parameters are gained from the total of subject's segments of the first minute of each task. The first minute of the tasks was chosen because it represents different workload conditions but without side effects like fatigue, exhaustion, or monotony. Although one could argue that the number of tasks is limited, we believe that it is sufficient to gain general information about the individual brain oscillation mode under different workload requirements.

**Figure 2:**
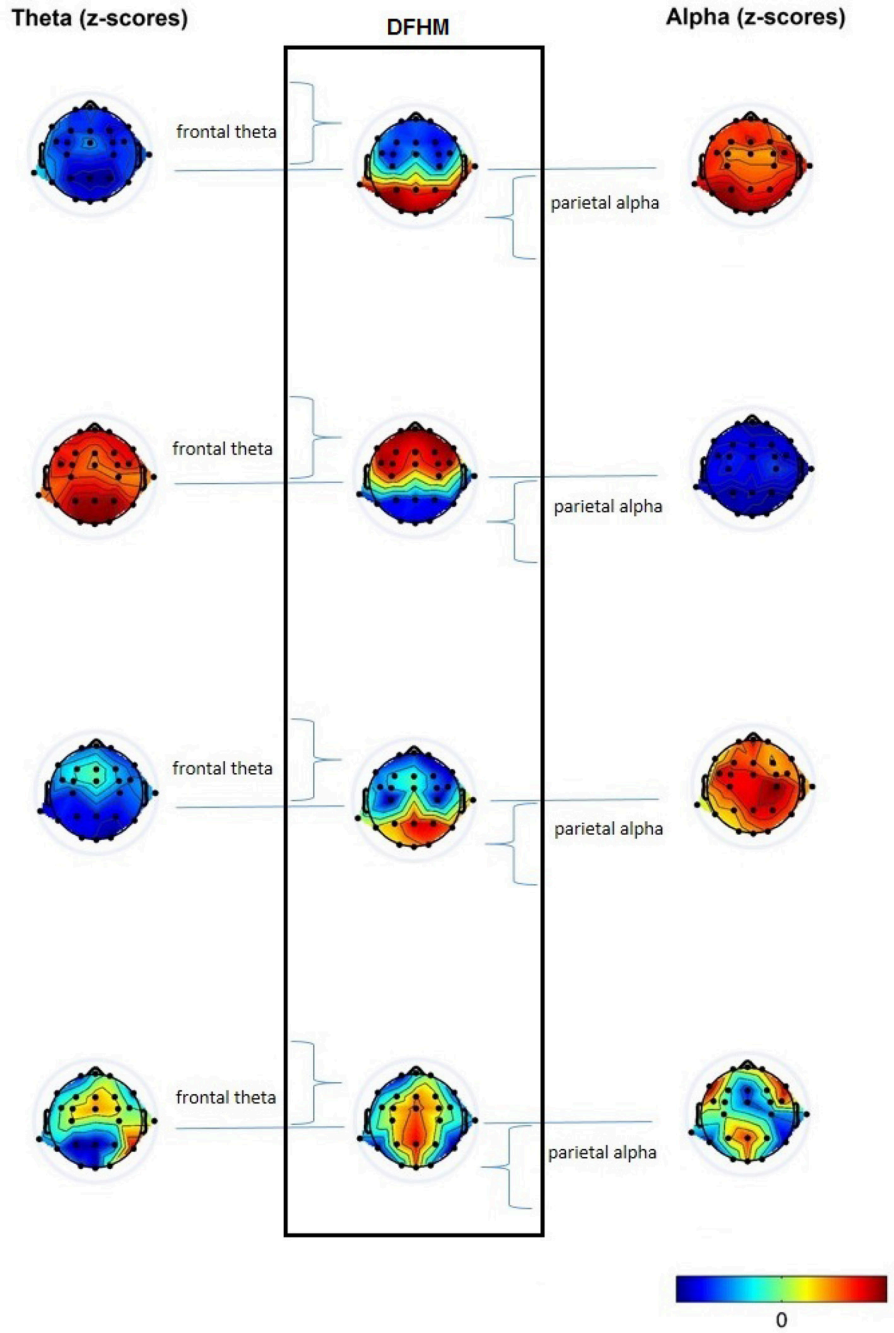
Generation of DFHM by combining the frontal theta band power with the parietal alpha band power in one head map.

Based on these individually computed four values for each electrode, we calculate for each person the z-scores for each segments' theta and alpha band power. Next, we collocate the z-scores of the theta band power at the frontal electrodes and the z-scores of the alpha band power at the parietal electrodes in one head map. This means that we comprise band powers from all electrodes, but include only the workload-relevant frequency bands in respect to the brain area. This compilation of the theta band power from the frontal electrodes and the alpha band power from the parietal electrodes leads to the DFHM for each EEG segment. Figure 2 shows how the DFHM are generated, and how the spatial information is kept.

#### 3.1.3. DFHM workload index

**Figure 3:**
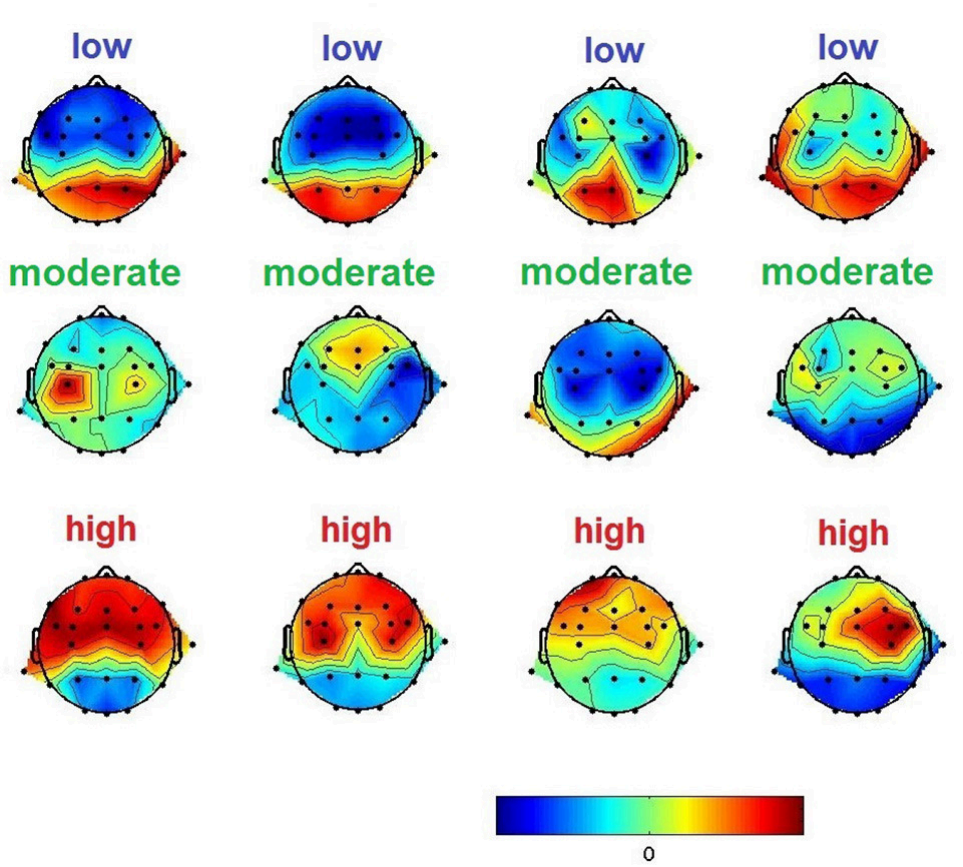
Examples of DFHM and their labels.

Based on long-term experience and in-depth knowledge of the literature in respect to the variability of the EEG depending on workload variance, we labeled 540 DFHM according to the well reputed behavior of the frequency band powers. The DFHM were selected from the first minute of each measurement (except of the AOSPAN task) of every subject (54 subjects × 10 measurements × 1 segment of the first minute). For the sake of consistency, we excluded the first minute of these tasks from the further proceeding of classifier test. The labels used represented the range of low load, moderate load, and high load. The labeling process was totally blind to subject and task and relied only on the behavior of the frequency bands. Accordingly, each DFHM was visually inspected. If the frontal z-score value of the theta band power was high and the parietal z-score value of the alpha band power was low, the DFHM was labeled as high load. In contrast, all DFHM with a low frontal z-score value and high parietal z-score value were labeled as low load. The remaining, where the differences of frontal and parietal z-scores were not pronounced, were labeled as moderate load. Figure 3 shows examples of such DFHM and their labels.

Subsequently, training of two support vector machines (SVMs) for low load and high load DFHM classification was carried out. We performed random sub-sampling, also known as Monte Carlo cross-validation. We are aware that by application of this method not all DFHM of our labeled data set might be used. However, we are also aware of our quite small set size. Given this, we consider an advantage of this method that there is no dependency of subset split-size to the number of folds (e.g., k-fold cross validation).

We split our data into a training and a testing subset, whereby their size corresponded to 60 and 40% of the labeled DFHM, respectively. These random partitioning of the data was repeated ten times. For each split the classifiers were retrained from scratch to fit to the new training subset and predictive accuracy was computed by means of the testing subset. Finally, the results were averaged over the splits. The empirically selected parameters and the testing results of both SVM kernels are presented in Table 2. The least classification accuracy of both classifiers yielded 92.69%.

**Table 2:** Optimal parameters and accuracy rates for the SVM classifiers.

	**C: SVM regularization**	**σ: kernel width**	**Mean recognition rate (%)**
Low load DFHM	0.03	3	94.06
High load DFHM	0.03	0.3	92.69

**Figure 4:**
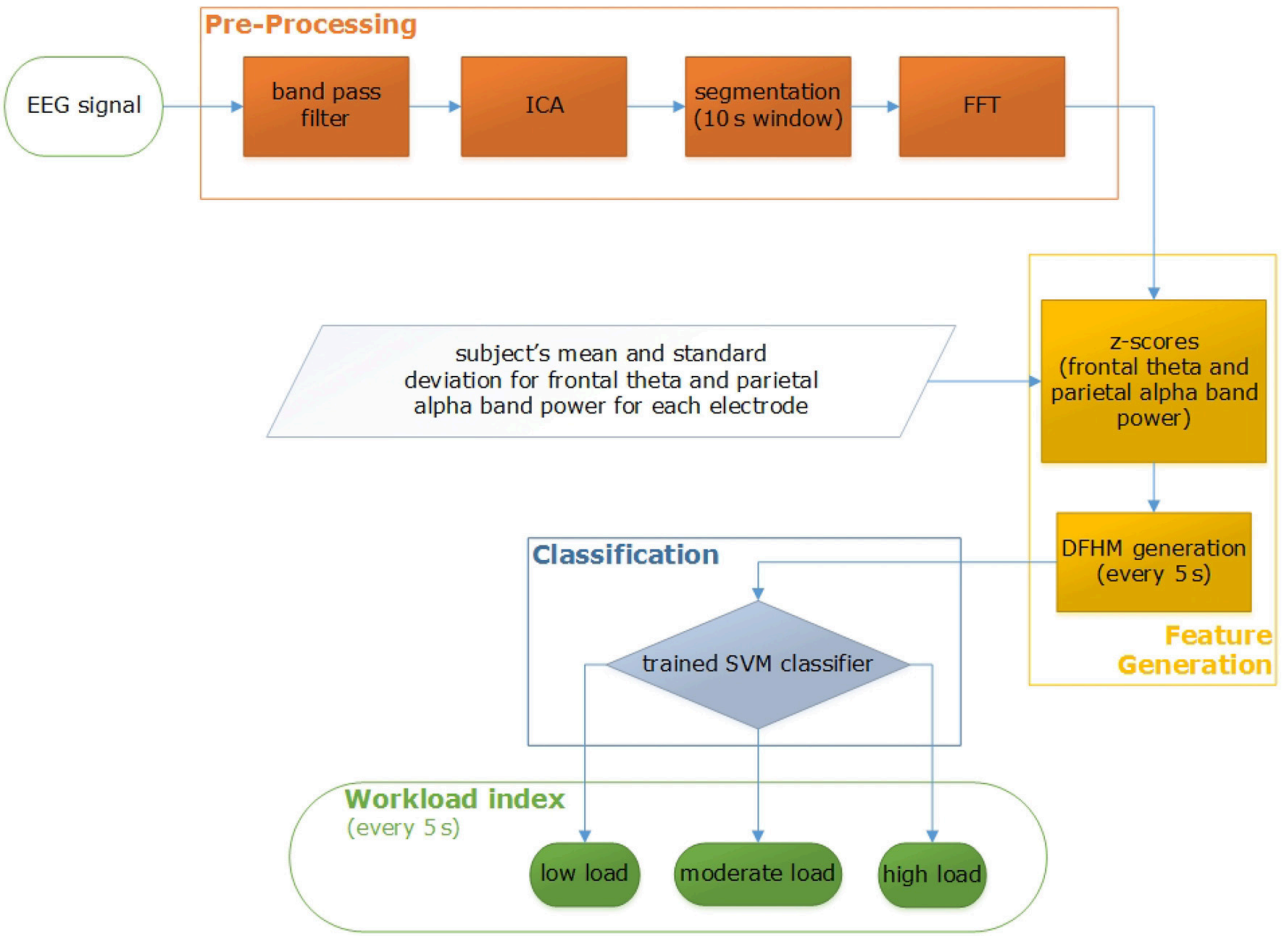
Processing pipeline for workload index determination.

Our system's setup consists of the general pre-processing of the recorded EEG data, computation of z-score and DFHM, and their classification. Figure 4 shows the processing pipeline.

**Figure 5:**
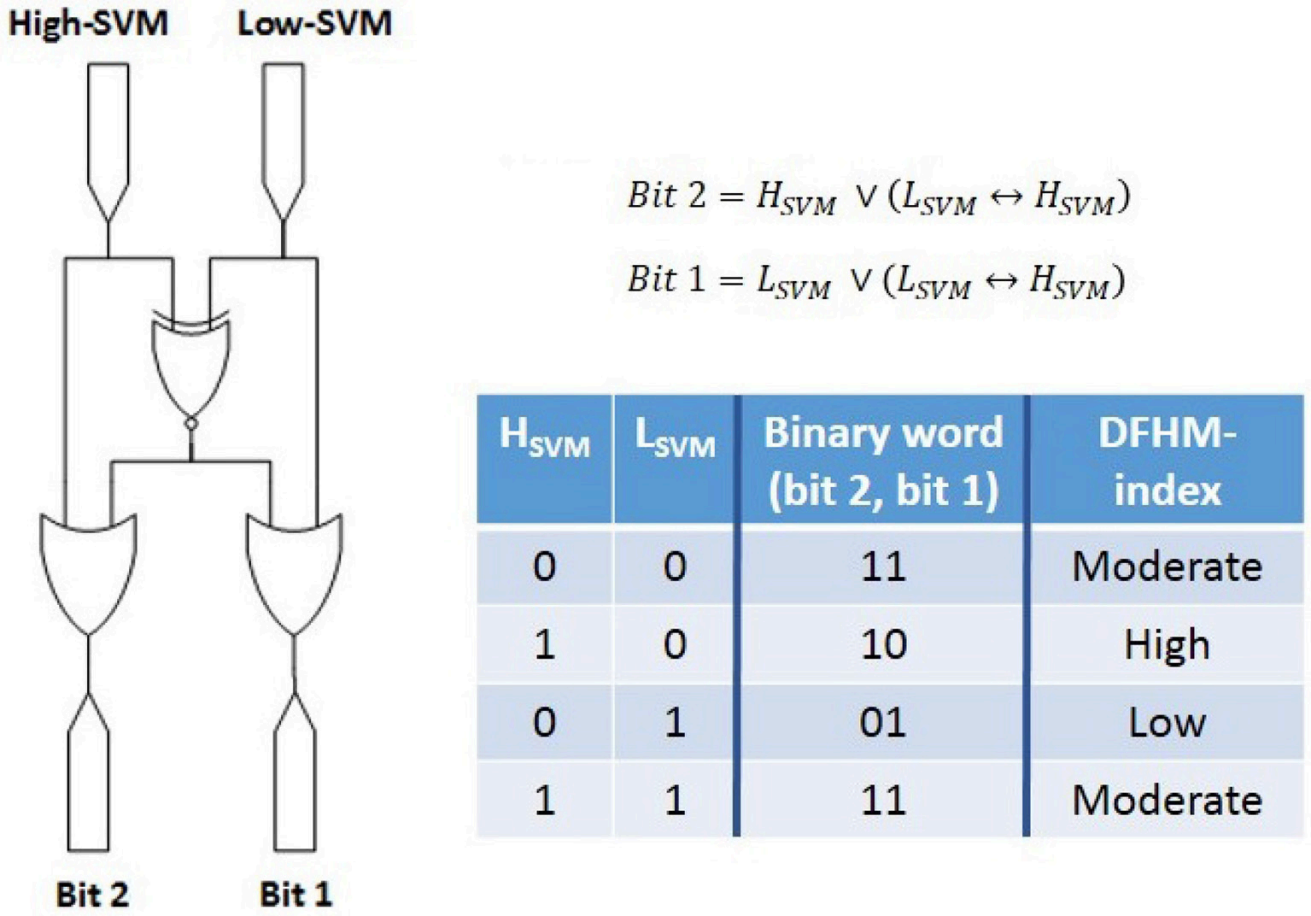
Computation of the new workload index as a logical combination of both SVM classifiers' output.

For every subject and measurement, DFHM are created of each EEG segment. Both SVM classifiers are applied on each DFHM and the new workload index is automatically computed as a logical combination of the classifiers' output every 5 s (Figure 5). Based on these index values all segments are classified as low, moderate, or high load segment.

**Figure 6:**
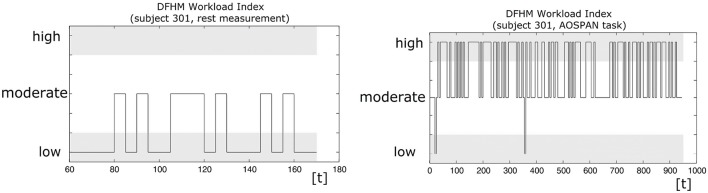
Examples of the DFHM workload index generated every 5 s: during the rest measurement at the beginning **(Left)** and during the demanding AOSPAN task **(Right)**.

In contrast to the single power spectra of the alpha and theta band there is no need anymore for defining quantitative threshold values for the three workload states. The DFHM achieve immediate classification in one of the three classes (Figure 6). An additional advantage is that labeling of the DFHM is task independent. Furthermore, the DFHM are also person independent due to the z-scores used. Hence, retraining of the classifier is not necessary—neither in respect to new subjects nor regarding new tasks. The only parameters needed relate to the calculation of the z-scores. They comprise the pre-saved information about the subject's mean and standard deviation of theta and alpha band power for each electrode.

### 3.2. Statistical analysis for evaluation of the DFHM workload index

It is quite obvious that labeling of our complete data set is not possible (1008 DFHM × 54 subjects). Hence, evaluation of the DFHM workload index is done statistically with regard to a postulated hypothesis. This arises from the cognitive task demands well-known from the literature and their impact on subject's mental workload. In particular, we expect easy tasks like 0-back, switch-NUM, switch-GER, and the rest measurements to induce low workload, i.e., relatively few DFHM segments classified as high load and most DFHM classified as low load. In contrast, high workload is expected during demanding tasks like AOSPAN. Here, most of the DFHM are expected to be classified as high load and only few of them as low load. Finally, the Stroop and the switch-XXX tasks are assumed to range in the middle and to induce moderate workload. Hence, the amount of DFHM classified as high and low load is expected to range between the above mentioned tasks.

For statistical evaluation of our new method, we calculated the percentage of segments in the three classes (low load, moderate load, and high load) getting three percentage values for each person and task. Thereafter, we computed two analyses of variance (ANOVA) with repeated measures design using the portion of low load segments (LLS) and the portion of high load segments (HLS) as within-subject factor, respectively. To complete the picture, we involved all measurements in our evaluation. Hence, two rest measurements and nine tasks constituted the eleven levels used. For the examination of significant differences between these levels, we employed *post-hoc* tests. Thereby, multiple paired comparisons with a Bonferroni correction were calculated. The Bonferroni correction is used to counteract the problem of multiple comparisons by adjusting the significance level by the number of the calculated comparison tests (Abdi, 2007). The correction aims to keep the false-positive error at 5% overall. Statistical analysis was computed by means of the software package SPSS.

Furthermore, regarding the NASA-TLX and the accuracy rates we calculated one more repeated measures ANOVA, respectively. They both comprised one within-subject factor and only nine levels (the nine tasks but no rest measurements). The ANOVAs were completed by Bonferonni-corrected *post-hoc* tests. These examinations allow us to compare the new workload index with further workload relevant data later on and gain additional information that could be beneficial in case of doubts when interpreting our DFHM results.

## 4. Results

**Figure 7:**
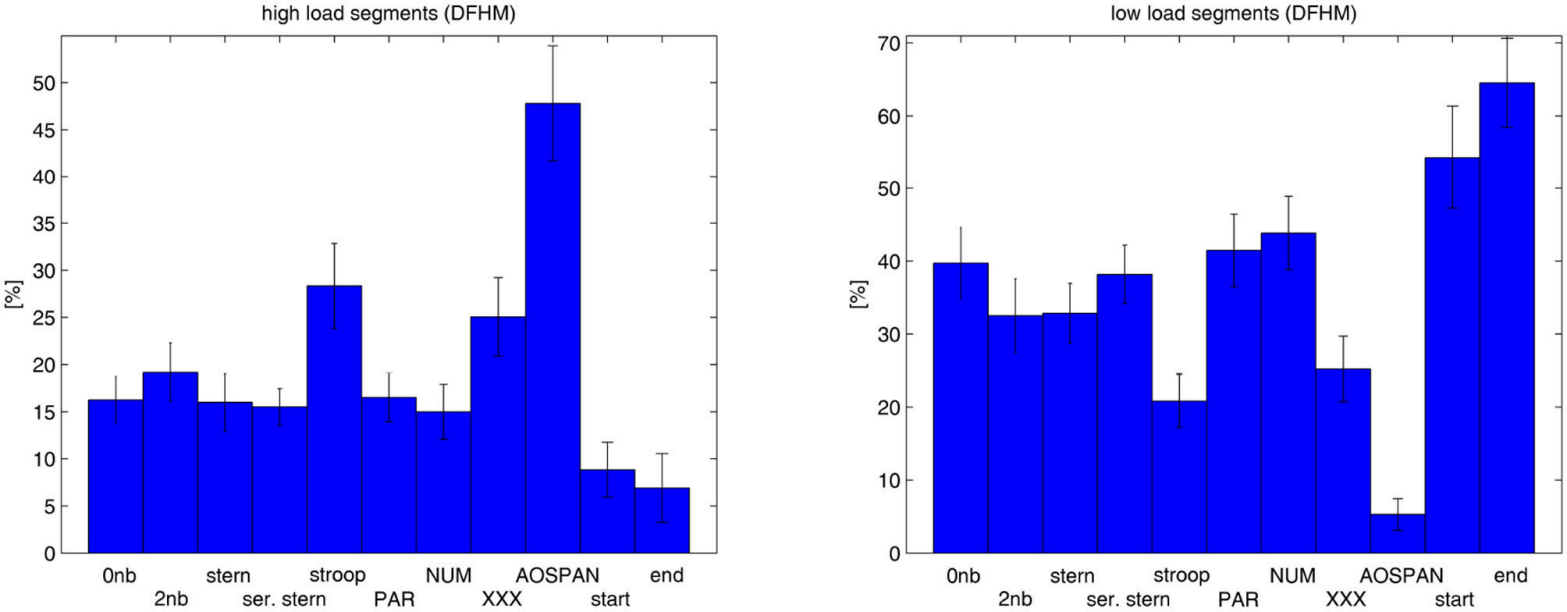
Portion of high load **(Left)** and low load segments **(Right)** computed for the rest measurements and the cognitive tasks over the subjects.

Statistical analysis yielded significant differences in the means of the portion of high and low load segments. These changes among the tasks were examined by *post-hoc* analysis. They are presented in Tables 3, 4 and illustrated in Figure 7. In particular, analyses show obvious differences between the easy tasks (rest measurements, 0-back, switch-PAR, and switch-NUM) and the more demanding ones (Stroop, switch-XXX, and AOSPAN) as well as between the most demanding AOSPAN task and the remaining. They are well pronounced for both: the proportion of high load and the proportion of low load segments but in different directions. Furthermore, we noticed the Stroop and the switch-XXX tasks to range in the middle between the easy tasks and the AOSPAN task. They have significantly higher proportions of high load segments than the easy tasks but significantly lower than the most demanding task. The proportion of low load segments behaves accordingly in the opposite direction. In general, we note that an increase of task demands leads as expected to an increase of the portion of high load segments while the portion of low load segments decreases.

**Table 3:** Significance thresholds for the discrimination of the portion of low load segments between the cognitive tasks based on the DFHM method [Greenhouse-Geisser: *F*_(5.57; 295.02)_ = 46.196, *p* < 0.001; *post-hoc* tests: ^***^: *p* < 0.001; ^**^: 0.001 ≤ *p* < 0.01; ^*^: 0.01 ≤ *p* < 0.05].

	**0nb**	**2nb**	**stern**	**s.ste**	**str**	**par**	**num**	**xxx**	**aos**	**sta**	**end**
0nb	–				^***^			^**^	^***^	^*^	^***^
2nb		–			^*^				^***^	^**^	^***^
stern			–		^***^		^*^		^***^	^***^	^***^
s.ste				–	^***^			^**^	^***^	^*^	^***^
str	^***^	^*^	^***^	^***^	–	^***^	^***^		^***^	^***^	^***^
par					^***^	–		^***^	^***^		^***^
num			^*^		^***^		–	^***^	^***^		^**^
xxx	^**^			^**^		^***^	^***^	–	^***^	^***^	^***^
aos	^***^	^***^	^***^	^***^	^***^	^***^	^***^	^***^	–	^***^	^***^
sta	^***^	^***^	^***^	^***^	^***^			^***^	^***^	–	
end	^***^	^***^	^***^	^***^	^***^	^***^	^***^	^***^	^***^		–

**Table 4:** Significance thresholds for the discrimination of the portion of high load segments between the cognitive tasks based on the DFHM method [Greenhouse-Geisser: *F*_(5.43; 287.99)_ = 41.58, *p* < 0.001; *post-hoc* tests: ^***^: *p* < 0.001; ^**^: 0.001 ≤ *p* < 0.01; ^*^: 0.01 ≤ *p* < 0.05].

	**0nb**	**2nb**	**stern**	**s.ste**	**str**	**par**	**num**	**xxx**	**aos**	**sta**	**end**
0nb	–				^**^				^***^	^*^	^**^
2nb		–							^***^	^**^	^***^
stern			–		^***^			^*^	^***^		^*^
s.ste				–	^***^			^*^	^***^	^*^	^**^
str	^**^		^***^	^***^	–	^**^	^***^		^***^	^***^	^***^
par					^**^	–		^**^	^***^	^**^	^**^
num					^***^		–	^**^	^***^		
xxx			^*^	^*^		^**^	^**^	–	^***^	^***^	^***^
aos	^***^	^***^	^***^	^***^	^***^	^***^	^***^	^***^	–	^***^	^***^
sta	^*^	^**^		^*^	^***^	^**^		^***^	^***^	–	
end	^**^	^***^	^*^	^**^	^***^	^**^		^***^	^***^		–

**Figure 8:**
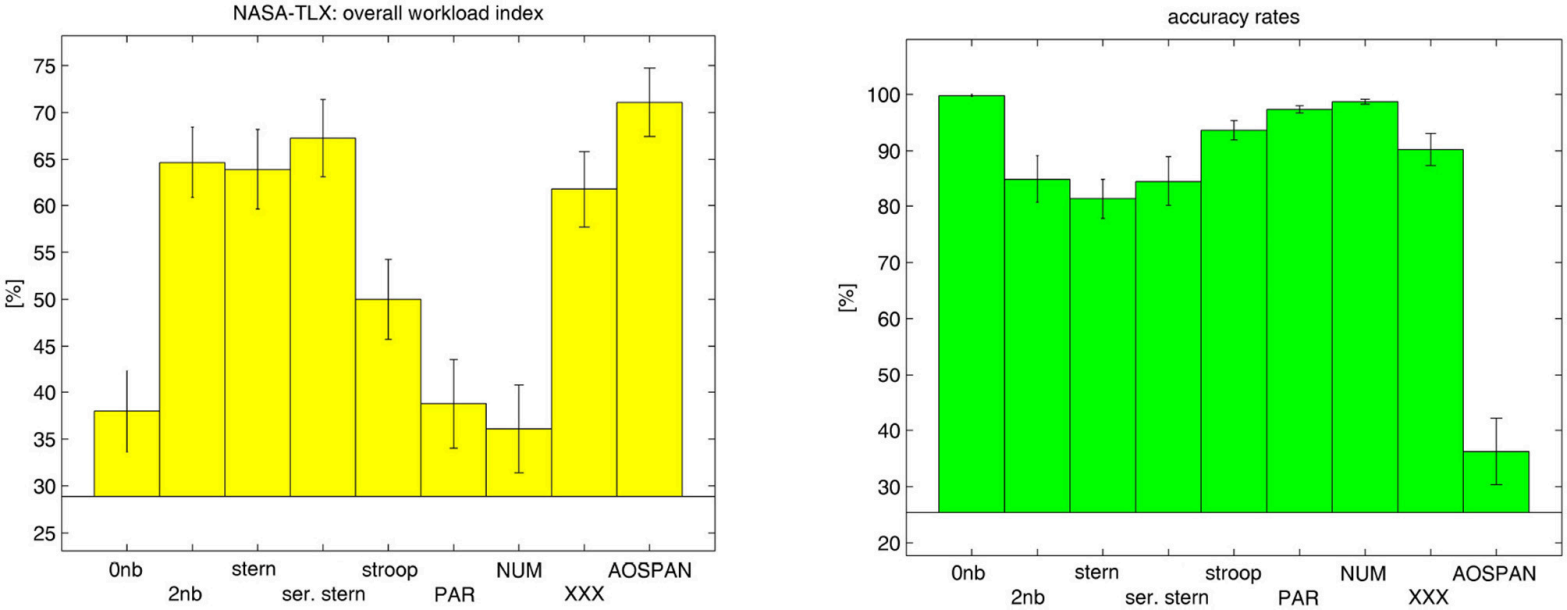
NASA-TLX overall index **(Left plot)** and accuracy rates **(Right plot)** computed for the nine tasks over the subjects.

Results of the subjective ratings and the accuracy rates computed for the nine cognitive tasks of the test battery over 54 subjects are illustrated in Figure 8. The left plot shows the average NASA-TLX workload index of each task. Analysis of variance and *post-hoc* tests showed significant changes of the mean workload index between most of the tasks (Table 5). The 0-back, switch-PAR, and switch-NUM tasks are subjectively rated as easy tasks by the subjects (workload index ≈ 37.6, NASA-TLX range: 0–100) and do not show any significant differences between each other. The Stroop task is rated as moderate (workload index ≈ 50), while the rest of the tasks show higher workload indices (workload index ≈ 65.8). Noteworthy is that the subjects did not differentiate between the five in respect to the cognitive demands quite differing tasks (i.e., 2-back, Sternberg, serial Sternberg, switch-XXX, AOSPAN) regarding the experienced workload. Furthermore, we noticed that the Stroop task shows significant differences to all other tasks.

**Table 5:** Significance thresholds for the discrimination of task load between the cognitive tasks based on NASA-TLX [Greenhouse-Geisser: *F*_(5.96; 316.01)_ = 65.02, *p* < 0.001; *post-hoc* tests: ^***^: *p* < 0.001; ^**^: 0.001 ≤ *p* < 0°.01; ^*^: 0.01 ≤ *p* < 0.05].

	**0nb**	**2nb**	**stern**	**s.ste**	**str**	**par**	**num**	**xxx**	**aos**
0nb	–	^***^	^***^	^***^	^***^			^***^	^***^
2nb	^***^	–			^***^	^***^	^***^		
stern	^***^		–		^***^	^***^	^***^		^***^
s.ste	^***^			–	^***^	^***^	^***^		
str	^***^	^***^	^***^	^***^	–	^***^	^***^	^**^	^***^
par		^***^	^***^	^***^	^***^	–		^***^	^***^
num		^***^	^***^	^***^	^***^		–	^***^	^***^
xxx	^***^				^**^	^***^	^***^	–	^*^
aos	^***^		^***^		^***^	^***^	^***^	^*^	–

The average accuracy rates are presented in the right plot. During the experiment, the means of the accuracy rates changed significantly. These significant changes were revealed by *post-hoc* analysis (Table 6) and show obvious performance differences between the easy tasks (0-back, switch-PAR, and switch-NUM) and the rest as well as between the most demanding AOSPAN task and the remaining. Furthermore, we noticed the Stroop task to have significantly higher accuracy rates than the working memory tasks 2-back, Sternberg, and serial Sternberg.

**Table 6:** Significance thresholds for the discrimination of accuracy rates between the cognitive tasks [Greenhouse-Geisser: *F*_(3.71;196.67)_ = 173.27, *p* < 0.001; *post-hoc* tests: ^***^: *p* < 0.001; ^**^: 0.001 ≤ *p* < 0.01].

	**0nb**	**2nb**	**stern**	**s.ste**	**str**	**par**	**num**	**xxx**	**aos**
0nb	–	^***^	^***^	^***^	^***^	^***^	^**^	^***^	^***^
2nb	^***^	–			^**^	^***^	^***^		^***^
stern	^***^		–		^***^	^***^	^***^	^***^	^***^
s.ste	^***^			–	^**^	^***^	^***^		^***^
str	^***^	^**^	^***^	^**^	–	^**^	^***^		^***^
par	^***^	^***^	^***^	^***^	^**^	–	^**^	^***^	^***^
num	^**^	^***^	^***^	^***^	^***^	^**^	–	^***^	^***^
xxx	^***^		^***^			^***^	^***^	–	^***^
aos	^***^	^***^	^***^	^***^	^***^	^***^	^***^	^***^	–

## 5. Empirical validation of the DFHM workload index by means of new experiments

For the empirical validation of the DFHM workload index we conducted a new experiment. Hereby, we tested an additional sample set of eight subjects (5 female, 3 male, age between 29 and 60 years, mean age 40). The new data were collected under the same conditions and procedure as the above mentioned experiment. In order to test if the new method can deal with new tasks without adjustment, we enlarged our task battery by an additional cognitive task: the stop signal task (Logan, 1994; Dimoska, 2005).

The stop signal task is similar to the Stroop task an inhibition task. During the task, subjects are instructed to press the green mouse button as fast as possible if a horizontal left arrow is presented on the screen and the red mouse button if a horizontal right arrow appears. If a horizontal arrow is shortly followed by a vertical arrow, they are instructed to inhibit their response and not press either button. They have to respond as fast as possible and consider that their main aim is to keep the frame around the arrow green. A red frame means they are too slow. Hence, if red, they should speed up their response while still paying attention to the vertical arrow.

For the validation we postulate the same hypotheses as above. If the method is valid, we have to obtain similar tendencies in the behavior of the proportion of high and low load segments among the tasks as for the first experiment. Furthermore, we expect that the new task yields similar results as the Stroop task. Finally, we have to highlight that SVM classifiers for the DFHM were not retrained for the new experiment, neither regarding the new sample set nor in respect to the new task.

**Figure 9:**
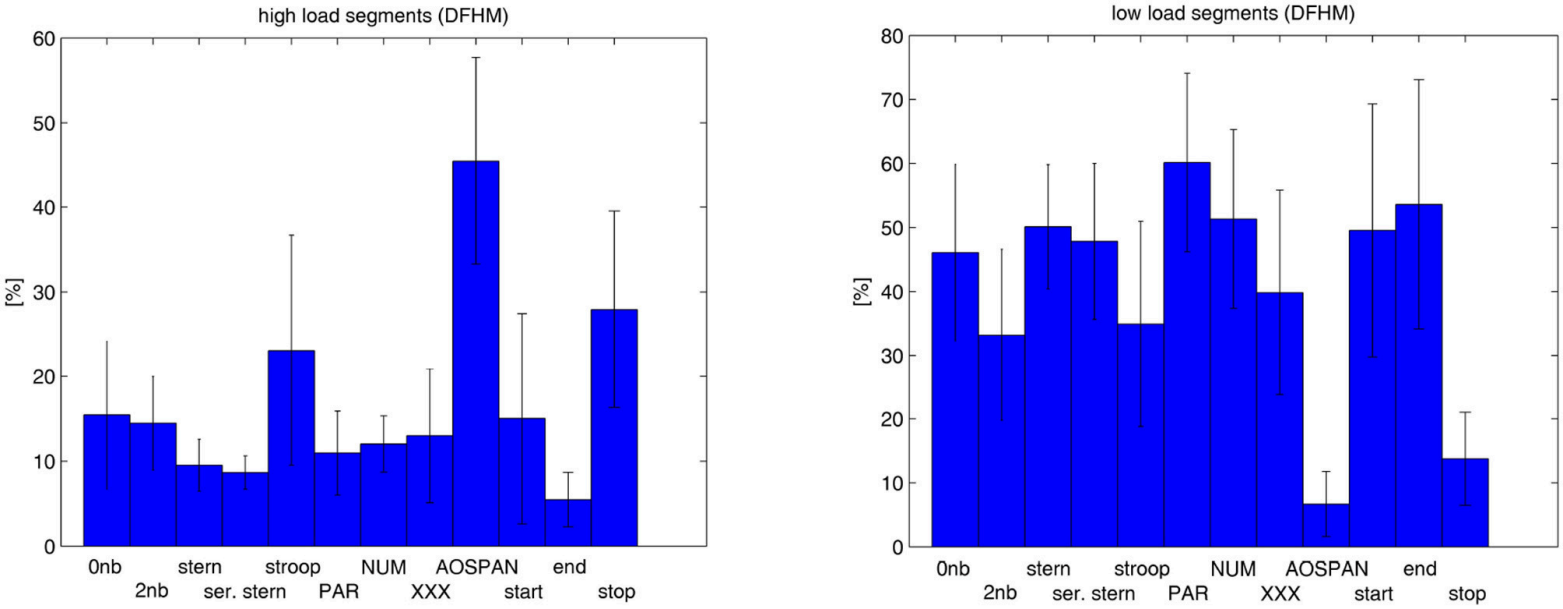
Empirical validation of the method based on a smaller sample set and an additional task: Proportion of high load **(Left)** and low load segments **(Right)** computed for the rest measurements, the nine cognitive tasks, and the additional stop signal task over a sample set of eight subjects.

The proportion of HLS and LLS computed over the eight subjects for the nine tasks and the two rest measurements are presented in Figure 9. We note here the same trends as observed previously for the larger sample set. What is more, the stop signal task is placed between the most demanding AOPSAN task and the Stroop inhibition task. Remarkable is its small proportion of LLS in respect to the Stroop task. However, no exploratory data analysis was carried out due to the small sample set.

## 6. Discussion and conclusions

In order to evaluate the new proposed DFHM method, in the following we consider the obtained results and discuss them also in comparison with the tendencies derived from the subjective ratings and the performance measurements.

Regarding the subjectively experienced mental workload derived from the NASA-TLX overall index no significant differences could be identified among the easy tasks 0-back, switch-PAR, and switch-NUM. This was also not expected because these tasks do not differ in respect of their minimal demands. Similarly, no significant differences among these easy tasks could be found in respect to the proportions of high and low load segments derived from the DFHM method.

Furthermore, analysis of the proportion of identified high and low load segments per task indicates that with increasing demands the amount of high load segments increases while the amount of segments classified as low load decreases. Moreover, the resulting DFHM workload index yields here the same tendencies as the other workload parameters from the questionnaire and the performance data.

It was somewhat surprising that the subjects were not able to significantly discriminate mental workload among the five tasks 2-back, Sternberg, serial Sternberg, switch-XXX, and AOSPAN which obviously differ in their task demands. We presume that there is a ceiling effect in the subjectively measured workload. As the workload reaches the subjective ceiling, the desired validity of the subjective ratings suffers. Interestingly, subjective ratings indicate significantly less workload during the Stroop task than during the five above mentioned tasks. We attribute this to the playful character of the Stroop task that could function as motivation and also lead to significantly higher performance compared to the working memory tasks 2-back, Sternberg, and serial Sternberg. However, the index derived from the DFHM places the Stroop task regarding mental workload between the demanding AOSPAN and the working memory tasks and on a similar level as that from the switch-XXX task.

Subsequently, we empirically validated the new method on a smaller sample set of eight new subjects and with one additional new task (the stop signal task). We want to emphasize that SVM classifiers were not retrained neither in respect to the new subjects nor to the new task. Validation of the DFHM workload index indicated the same tendencies among the tasks as already obtained by the larger sample set. The additional stop signal task integrated itself as expected between the demanding AOSPAN task and the Stroop inhibition task. Noticeable is that in respect of the proportions of LLS from the Stroop and the stop signal task, the latter is found to be more demanding. This fact fits well to the statements of the subjects regarding their difficulties during the stop signal task.

To sum up, the new DFHM method leads to the classification of EEG segments as high, moderate, or low load. Based on this, our results support the expected difficulty levels of the tasks resulting from their demands on the executive functions.

In contrast to single power spectra (e.g., alpha and theta band) there is no need anymore for defining quantitative threshold values for the three workload states. Threshold values are problematic because they are not generally applicable to different subjects. As the new method relies on personalized DFHM and on SVM classifiers trained by means of reported knowledge on the variability of spectral features, it achieves immediate and continues classification in one of the three classes. The time-consuming, additional retraining of the classifier for new subjects and tasks is not necessary anymore. Results from the other workload relevant parameters (subjective ratings and performance data) reveal similar outcomes regarding workload and emphasize the EEG findings.

Based on such consolidated findings of neuronal brain states an optimal workload range for the operator can be defined. This range corresponds to more efficient cognitive processing, thus enabling performance at its best. Simultaneously, it preserves employee's health and hence, accounts for optimized work conditions.

Related research areas are also neuroergonomics (Parasuraman and Rizzo, 2008), augmented cognition (Schmorrow et al., 2009), and physiological computing (Fairclough, 2009; Fairclough and Gilleade, 2014). The main goal of such research is to use continuous information about the individual's mental state for improving task sharing between human and machine. Our approach to develop a method for continuous mental workload registration contributes to these research areas. The overall important benefit is the prevention of negative impacts of sustained high or low load on the mental health and cognitive capacity of the working population. Brain state monitoring can contribute to the modulation of workload, protect and advise against high load and low load, and can be used for ergonomic evaluation and improvement of human-machine systems and information intensive occupations.

As a limitation of our work, we have to mention that our experiment is conducted in a highly controlled laboratory environment by using clearly defined, basic cognitive tasks. Results could be different under realistic settings and real world scenarios. Especially, the contradictory behavior of the alpha band power could effect the validity of the DFHM workload index under realistic conditions with enhanced visual information processing. Further research is needed to validate these findings in such environments. The first steps for research outside the lab, in particular regarding automatic artifact rejection, have already been taken (Radüntz et al., 2015, 2017). In addition, further research is needed to prove if aggregation of the DFHM workload index over time is more appropriate regarding feasible state changes. Taken into account that cognitive states are global, we have to ask if an interpretation of cognitive states on a second-by-second basis is reasonable. Hence, apart from the question of the best suitable aggregation type, the question of an adequate time window should be addressed, too. These still outstanding research issues will be addressed within the framework of our current project with air traffic controllers and in cooperation with the German Aerospace Center.

## Author contributions

TR implemented the task battery and was responsible for all technical and computational work of the study. Signal processing, data analysis, and interpretation were performed by TR. The manuscript was written by TR.

### Conflict of interest statement

The author declares that the research was conducted in the absence of any commercial or financial relationships that could be construed as a potential conflict of interest.
